# Perceptions of important outcomes of moral case deliberations: a qualitative study among healthcare professionals in childhood cancer care

**DOI:** 10.1186/s12910-021-00597-4

**Published:** 2021-03-17

**Authors:** Charlotte Weiner, Pernilla Pergert, Bert Molewijk, Anders Castor, Cecilia Bartholdson

**Affiliations:** 1 Childhood Cancer Research Unit, Department of Women’s and Children’s Health, Karolinska Institutet, Stockholm, Sweden; 2 Paediatric Regional Health Care, Astrid Lindgren Children’s Hospital, Stockholm, Sweden; 3 Paediatric Haematology and Oncology, Astrid Lindgren Children’s Hospital, Stockholm, Sweden; 4 Department of Medical Humanities, Amsterdam University Medical Centers, Location VUmc, Amsterdam, The Netherlands; 5 Center for Medical Ethics, University of Oslo, Oslo, Norway; 6 Paediatrics, Lund University, Lund, Sweden; 7 Pediatric Oncology, Hematology, Immunology and Nephrology, Skåne University Hospital, Lund, Sweden; 8 Paediatric Neurology and Musculoskeletal Disorders and Homecare, Astrid Lindgren Children’s Hospital, Stockholm, Sweden

**Keywords:** Childhood cancer care, Clinical ethics, Clinical ethics support, Healthcare professionals, Moral case deliberations, Moral challanges, Outcomes, Qualitative

## Abstract

**Background:**

In childhood cancer care, healthcare professionals must deal with several difficult moral situations in clinical practice. Previous studies show that morally difficult challenges are related to decisions on treatment limitations, infringing on the child's integrity and growing autonomy, and interprofessional conflicts. Research also shows that healthcare professionals have expressed a need for clinical ethics support to help them deal with morally difficult situations. Moral case deliberations (MCDs) are one example of ethics support. The aim of this study was to describe the MCD-related outcomes that healthcare professionals in childhood cancer care considered important, before MCDs were implemented, in order to facilitate the implementation of MCDs in childhood cancer care in Sweden.

**Methods:**

This study is based on qualitative data. Healthcare professionals, mostly representing registered nurses, nursing assistants and physicians, working at childhood cancer care centres in Sweden, were invited to respond to the translated and content validated European MCD Outcomes Instrument, before participating in regular MCDs. Answers to the main open-ended question, included in the questionnaire, was analysed according to systematic text condensation.

**Results:**

Data was collected from 161 responses from the healthcare professionals. The responses included healthcare professionals’ perceptions of which MCD-related outcomes they found important for handling moral challenges. Three different themes of important outcomes from the analysis of the data are presented as follows: *Interprofessional well-being in team interactions* on a team level; *Professional comfort when dealing with moral challenges* on a personal level; and *Improved quality of care for the child and the family* on a care level.

**Conclusions:**

Healthcare professionals in childhood cancer care considered it important that ethics support could enhance the well-being of interprofessional teams, support healthcare professionals on an individual level and improve quality of care. The results of this study can be used in current and future training for MCD-facilitators. When knowing the context specific important MCD-outcomes, the sessions could be adapted. Managers in childhood cancer care would benefit from knowing about the specific important outcomes for their target group because they could then create relevant working conditions for clinical ethics support.

## Background

Clinical ethics support services (CESS) offer various ways of supporting healthcare professionals (HCPs) to deal with the moral challenges involved in their work [1]. CESS could help interprofessional teams to stimulate ethics reflection and to handle moral challenges. One example of CESS is moral case deliberations (MCDs), also referred to as ethics case reflection rounds [2], which involve reflection by the interprofessional team on specific moral challenges in clinical cases [3, 4]. In MCDs, a structured conversation method is used by a trained and certified facilitator [5] focusing on moral issues such as what is morally right to do and how should this be done in a right way [4].

In Sweden, approximately 300 children are diagnosed with cancer each year [6]. Childhood cancer care has seen a dramatic development during the last century, so that today more than 80% of the children in high-income countries are cured [7]. The high survival rate is the result of often complex treatment regimens given over months to years. The treatments can be quite intensive and burdensome, and for the most advanced cancers also life threatening. Despite the very encouraging survival rates, 15–20% of the children still die, most often due to refractoriness or recurrence of the cancer, or due to the toxicity of the treatment itself. Caring for children who are suffering from life-threatening illnesses can lead to emotional and psychological stress, including associated moral challenges, for HCPs [8, 9]. Previous research has also shown that in some settings, moral challenges occur more often in paediatric care [10]. In childhood cancer care as in many other medical contexts, advanced medical, supportive and nursing care is provided, and the work environment is often described as stressful, with insufficient staffing and heavy workloads [8, 9]. Hence, working within highly specialised childhood cancer care undoubtedly involves being confronted with various moral challenges and complex decisions where important values are at stake [11]. Besides that, HCPs often need to consider different and sometimes opposing views on the part of the interprofessional team, the family and the child when determining the best care [11–13]. Thus, a triad of stakeholders are usually involved in assessing a child’s best interest.

HCPs in paediatric settings, including childhood cancer care, face many moral challenges such as infringing on the child's integrity and growing autonomy when deciding on the best care; for example, by not telling the truth about poor prognoses or performing procedures against the child’s will [11, 14]. Other moral challenges in decision making may be due to differing views on treatment levels [11, 15]. Lack of interprofessional interaction and different perspectives on end-of-life care may sometimes lead to insoluble conflicts and social tensions [16], both within the team and on an individual level. Conflicting interprofessional viewpoints on what good care entails can lead to moral distress [8]. Moral distress is often observed among HCPs as they contribute to avoidable harm when acting, not acting or deciding against their own values, on account of internal or external constraints [17–19]. Previous research demonstrates that moral distress can be reduced by strengthening HCPs’ moral courage [20–22]. Other strategies for reducing moral distress include encouraging HCPs’ ability to overcome fear by confronting issues that conflict with their professional values [23] and sharing negative emotions constructively within the team [24]. HCPs have expressed a need for an organisational structure that can support them in dealing with moral challenges and moral distress, enabling a more systematic, constructive and effective ethical reflection at their workplace [25]. This need has also been highlighted in a study from highly specialised childhood cancer care [26].

Several studies have shown that implementing MCDs as a specific kind of CESS can help HCPs to better deal with moral challenges [4, 26, 27]. However, before implementing MCDs, it is important to find out about HCPs’ goals and expectations regarding MCDs, and what support and outcomes they are hoping to achieve in order to obtain more specific guidance in handling moral challenges. Although there is a recent study on what outcomes MCD participants find most important in adult settings [28], there is a lack of such research in the field of paediatrics, as well as in childhood cancer care. In connection with implementation of MCDs at the six childhood cancer centres in Sweden, the opportunity was given to explore what outcomes HCPs perceived as important in order to ensure, understand and develop existing CESS. Therefore, this study was initiated, using the answers to an open-ended question in the European Moral Case Deliberation (Euro-MCD) Outcomes Instrument [26].

## Aim

The aim of this study was to describe the MCD-related outcomes that HCPs in childhood cancer care considered important, before MCDs were implemented, in order to facilitate the implementation of MCDs in childhood cancer care in Sweden.

## Methods

This is a descriptive study based on a qualitative systematic analysis of written answers from the open-ended question included in the Euro-MCD [29].

### Data collection

Data were collected between September and December 2017 using a pen-and-paper questionnaire. Most of the responses were quite detailed and extensive; only a few responses consisted of single words. Participants wrote descriptively about MCD outcomes they considered important to reach. In this study, outcomes are defined as results of MCDs achieved during or after the MCDs.

#### The Euro-MCD instrument

The Euro-MCD is a multi-item instrument [29] that has been translated and culturally adapted to Swedish, Norwegian and Dutch from the original English version. The instrument contains an open-ended question and 26 predefined specific items representing various MCD outcomes. Participants are asked not to read the predefined items before answering the open-ended question. Furthermore, the Euro-MCD consists of two parts: the first to be answered prior to participation in MCDs and the second to be answered after participation [29]. This study is based on answers to the open-ended question in the first part *prior* to participation in MCDs. The question was worded as follows: “Imagine participating in MCDs. Please formulate in your own words three to five outcomes that you consider important to achieve in order to support you and your co-workers in handling moral challenges in everyday clinical practice”.

#### Study participants

National training of facilitators (N = 15) of MCDs had recently been conducted, involving professionals from all six paediatric cancer centres in Sweden. The trainee facilitators were planning to implement MCDs at their centres during the training. The trainee facilitators assisted the research group with data collection for this study before implementing MCDs. HCPs (n = 275) who worked clinically with childhood cancer patients and were presumptive participants in the upcoming MCDs were invited to participate in the study.

### Data analysis

The handwritten answers were transcribed to digital documents by the first and last authors. The analysis was inspired by Malterud’s (2012) modified systematic text condensation (STC) and included continuous self-reflection with regard to personal preconceptions about the data [30]. According to Malterud [31], STC is a method for analysis of qualitative data developed from traditions shared by several methods for qualitative analysis. This methodology offers the researcher a process of feasibility, reflexivity and intersubjectivity while sustaining a responsible level of methodological rigour [31]. STC was chosen because of its suitability for analysis of data collected through open-ended survey questions and for research that studies human expectations [30, 31]. The analysis was performed gradually over four phases: (1) overall impression; (2) identification of meaning units; (3) abstraction of the content; and (4) text summarising [31]. In the first phase, data were read repeatedly, carefully and reflectively, maintaining an open mind [31, 32]. This provided an idea of what the data entailed, and preliminary themes were written down, for example: *“Team collaboration”*. In the second phase, data were divided into meaning units. A manual analysis was performed, and the meaning units were separated and coded to form preliminary sub-themes, for example: *“Shared communication”*. Meaning units and themes were compared with each other. The process also involved exclusion of unclear text, i.e. where only one word was written [31]. In the third phase, a deeper analysis was performed, and the themes were given names describing what was meant by the meaning units. For example, the meaning unit “A [care] plan for the patient that all HCPs work towards” was coded to the preliminary subtheme “A common care plan”. In phase four, subthemes and quotations were translated from Swedish to English, and the emerging themes and results were summarised and finalised by assessing the findings in relation to the transcripts as a whole [31]. An example of the process of analysis is shown in Table 1. The themes and subthemes were discussed throughout with all co-authors in relation to the data.

**Table 1:** A sample of meaning units, subthemes and themes to exemplify the process of analysis

Meaning unit	Sub-themes	Theme
*“Improved communication that helps to define various aspects of the ethical problem and gain understanding and insight into the opinions of others.”*	Interprofessional understanding	Interprofessional well-being in team interactions
*“MCDs give us in the interprofessional team the opportunity to look at a situation from different perspectives and understand different ways of viewing/handling the situation.”*		
*“Open and permissive atmosphere in the group, where everyone can be given space to express themselves.”*	Permissive dialogue	
*“More communication between professionals. You dare to discuss and say what you think.”*		

## Results

The participants represented eight different professions: physician, registered nurse, nursing assistant, priest, psychologist, social worker, sibling supporter and hospital play therapist. Responses were received from 185 out of 275 HCPs who had received a questionnaire, representing a response rate of 67%. Of the 185 returned questionnaires, 161 included answers to the open question.

The results will be presented in themes and subthemes. The themes include: *Interprofessional well-being in team interactions* on a team level; *Professional comfort when dealing with moral challenges* on a personal level; and *Improved quality of care for the child and the family* on a care level. Themes and subthemes are presented below (Table 2) and quotations are used to exemplify the subthemes.

**Table 2:** Overview of themes and subthemes

Themes	Subthemes
Interprofessional well-being in team interactions	Interprofessional understanding
	Interprofessional decision-making
	Permissive dialogue
	Being confirmed
Professional comfort when dealing with moral challenges	Moral and practical competence
	Self-awareness and coping
	Moral courage and confidence
Improved quality of care for the child and the family	Understanding of the family situation
	A common care plan
	Supporting the child and family

### Interprofessional well-being in team interactions

Participants described how MCDs could promote outcomes related to interprofessional well-being through positive interactions and a permissive atmosphere within the interprofessional team as it tackles moral challenges. Moreover, interprofessional well-being is promoted by mutual respect between different professionals, and by the team having an inclusive attitude. Interprofessional well-being in team interactions includes four subthemes: *Interprofessional understanding; Interprofessional decision-making; Permissive dialogue;* and *Being confirmed.*

#### Interprofessional understanding

This subtheme concerns *increased* mutual understanding within the interprofessional team, involving both understanding between different professions and within their own profession. HCPs stated that understanding was an important outcome that could be achieved by sharing perspectives, as well as receiving the views and opinions of others.

*“Increased understanding between individuals”* (registered nurse)

Some participants emphasised the importance of a coherent view among co-workers that could help promote a peaceful and safe interprofessional team. Interprofessional understanding was considered important not only to promote interprofessional well-being, but also to improve interprofessional decision-making.

#### Interprofessional decision-making

In this study, participants wrote that one important outcome of MCDs was to enhance their knowledge of profession-specific facts, such as medical care, nursing care and the family situation. This was considered to be information that could help facilitate the team’s decision-making. It was also important to be able to discuss the pros and cons of decisions and actions.

*“A place where we can discuss with each other and come to a decision”* (registered nurse)

Moreover, interprofessional decision making included ensuring that the same information and a common overview of the situation were available to all members of the team. Several participants stated that they expected to agree on how to deal with moral challenges and reach well-founded solutions.

*“Safer decision-making that the majority agrees on”* (registered nurse)

*“Well-founded decisions in difficult situations”* (physician)

Besides the importance of improved interprofessional decisions, HCPs stated the importance of a permissive team dialogue.

#### Permissive dialogue

HCPs in this study described the importance of facilitating “free space” where different perspectives, problems and opinions could be aired openly as an outcome of MCDs. This included allowing everyone to have their say, with the same rights and opportunities to express themselves within the team.

*“An open, permissive conversational climate”* (registered nurse).

*“Open discussion where everyone can have a say”* (physician)

Several participants described a desire for the team to tolerate, respect and accept the reasoning of others and to understand people’s different approaches.

*“To be able to discuss and voice the thoughts arising in ethically difficult situations”* (registered nurse)

Furthermore, participants expected to encounter an environment in which no-one was judged, and where they could be confirmed.

#### Being confirmed

Participants described being confirmed as an important MCD-related outcome, allowing them to better handle moral challenges in care. Being confirmed meant feeling that their own thoughts and opinions were respectfully acknowledged by the team and that their feelings, opinions and thoughts were not considered strange. In the words of one participant:

“*Confirmation that you are thinking along the right lines” (registered nurse)*

Confirmation of their reasoning was considered to be an important outcome, generating a sense of security regarding what was ethically right or wrong in a specific situation. Another important aspect of confirmation was feedback on how previous situations had been handled, or that a case was in fact “ethically challenging”.

### Professional comfort when dealing with moral challenges

Professional comfort when dealing with moral challenges involves increased feelings of competence and confidence and decreased feelings of anxiety. Professional comfort is also about feeling safe, secure and comfortable in handling moral challenges in clinical practice. It includes three subthemes: *Moral and practical competence; Self-awareness and coping;* and *Moral courage and confidence.*

#### Moral and practical competence

Increased moral and practical competence were viewed as important outcomes. Moral competence includes broadening the ability for moral reasoning and increasing the capacity for empathy. Participants found it important to develop analytical reasoning and to evaluate as well as prioritise between different ethical principles regarding treatment alternatives. Another aspect involved the development of creative reflection, which participants stated could potentially bring new perspectives and thoughts on moral challenges. Essentially, participants found it important to gain an understanding of the complexity of moral challenges. Moral and practical competence were also referred to in terms of the ability to quickly and easily understand whether a situation involves a moral challenge, and to identify the essence of what is actually difficult on a *moral* level.

*“An enhanced ability to discern different aspects of moral dilemmas. Enhanced understanding of other perspectives in respect of moral reasoning”* (physician)

Besides moral competence, practical competence was also considered important. Practical competence includes facilitating a professional approach and knowing how to do things. For example, how to communicate difficult and sad information and what information to convey to the child and their family. It was also considered important to be able to deal with crises and demanding situations such as poor prognoses and death.

*“Better equipped and more tools to handle ethically difficult situations”* (registered nurse)

*“Enhanced security in our profession. Broaden my expertise and increase preparation for potential scenarios in the care of our patients”* (registered nurse)

Self-awareness and coping were also given as important MCD outcomes in order to reach a certain level of professional comfort when dealing with moral challenges.

#### Self-awareness and coping

Participants expected MCDs to potentially lead to greater self-awareness. HCPs considered it important to improve their ability to understand themselves and their own opinions and perspectives, and to be aware of why they do what they do.

*“Understanding your own reactions and the reactions of others, greater self-awareness, greater acceptance of your own resources and abilities…”* (registered nurse)

It was important for participants to find peace of mind when decisions were made on challenging cases, even if such decisions were perceived to be unsatisfying. Moreover, participants referred to important outcomes that were related to self-control; for instance, improving their own deliberations before making decisions on situations.

Coping with moral challenges was considered to be an important outcome and was described as being able to handle and reduce feelings of inadequacy and moral distress. *“Reducing moral distress” (nursing assistant)*. Several participants indicated that they thought that MCDs could help reduce stress in challenging situations.

*“Reducing stress in relation to complex ethical conflicts; for myself, and within the professional team”* (physician)

Minimising challenging thoughts after work was also described as an important outcome by participants, helping them to cope, because the moral challenge has already been dealt with in the MCDs. HCPs also expressed that they felt it was important to develop moral courage and confidence.

#### Moral courage and confidence

There was an expectation that participating in MCDs could increase moral courage, both when caring for children and their families and when communicating within the team. Participants described moral courage as being brave and confident enough to speak up and support others to express their opinions on moral grounds, and to dare to argue in favour of them despite other HCPs’ values and opinions.

*“Giving me greater strength and supporting others to dare to express ethical considerations and possibly ‘differing opinions’”* (physician)

Participants declared that sharing their own and other HCPs’ experiences, thoughts and feelings in MCDs was a way of potentially enhancing self-confidence. Participants also said it was important to develop the courage and confidence to talk about difficult situations such as treatment levels, palliative care and the perspectives of the family and child.

*“Being braver to handle all kinds of situations”* (registered nurse)

*“Security in meeting people in difficult situations and in dealing with ethical dilemmas. To be able to meet people, to dare.”* (registered nurse)

It was about venturing to spend time with the child and their family in their vulnerable situation and being able to handle the family’s feelings of shock when they are told of a poor prognosis or death.

*“I would like to be able to handle a crisis in cases of poor prognosis and death”* (registered nurse)

#### Improved quality of care for the child and the family

Participants described how MCDs could promote the quality of care for the child and the family in several domains, which forms three subthemes: *Understanding of the family situation; A common care plan;* and *Supporting the child and family.*

#### Understanding of the family situation

HCPs described achieving a deeper understanding of the family situation when caring for a child with cancer as an important outcome. Understanding of the family included increased respect for the family’s opinions and seeing the child as a part of his/her family.

“*Greater understanding of how a child’s difficult situation affects the child’s own family” (social worker)*

This also relates to the ability to see and to understand differences in various families, described by one nursing assistant as *“seeing who you have in front of you”.* It was also described as important to understand the family situation throughout the whole care process and to take the best interests of the child and the family into account. Moreover, it was considered important for the team to develop a common care plan that the family also agreed with.

#### A common care plan

HCPs stated that one important outcome was to generate, together with the family, a distinct common care plan for the child which included what was best for the child.

*“Ensuring that both the HCPs and the family feel that the decisions made are right for the child”* (registered nurse)

*“Better care, with more thought going into it”* (registered nurse)

Other aspects involved maintaining good care for the child and their family, adhering to the care plan and facilitating the follow-up and evaluation of previous care initiatives.

*“Follow-up and evaluation of our choices and actions”* (registered nurse)

Some participants felt it was important that MCDs create a possibility for HCPs to be capable of planning for continuity of care, allowing the same HCPs to continue to care for the child and their family. Participants also thought that supporting the family was an important outcome, which could lead to better care for the child and their family.

#### Supporting the child and family

HCPs described the provision of better psychological and social support for both the child and their family as another important outcome of MCDs. Being supportive was described as giving the child and their family good and safe care, and particularly improving care for families in crisis.

*“Better care for families who are in shock”* (registered nurse)

*“Better care and support for the family”* (registered nurse)

Comforting and listening to families, and talking about difficult situations and moral challenges, were also seen as important outcomes out of MCDs. Also important was the ability to prepare children and their families for future care initiatives, as well as knowing when and how to perform caring procedures. Supporting the family also includes being better prepared for problematic relationships in care situations, such as mediating in conflict situations.

## Discussion

This study presents the responses of HCPs in childhood cancer care to an open-ended question about what MCD-related outcomes they hoped to achieve and considered important for handling moral challenges in healthcare situations. The outcomes are combined in three themes: *Interprofessional wellbeing in team interactions; Professional comfort when dealing with moral challenges;* and *Improved quality of care for the child and the family.* These themes relate to the team, the individual professional and care, respectively. In the following section, highlighted results are discussed and compared with the existing literature on CESS outcomes.

A study by Dauwerse et al. (2013) shows that participants thought that the goals of CESS included encouraging a moral climate, as well as developing professionalism and good care [33], which are similar to the themes presented in our study. The findings from a Dutch study [34] designed in a similar way to the present study describe themes both comparable and non-comparable to those in this study. The authors of that study present the theme of “Better teamwork”, including important outcomes such as “More open communication” and “Better mutual understanding”, in a similar manner to our theme “Interprofessional well-being in team interactions”, including “Permissive dialogue” and “Interprofessional understanding”. They also present the themes “Better dealing with ethically difficult situations” and “Becoming a better professional” in a similar way to our subthemes “Interprofessional decision-making” and “Moral and practical competence” in this study. However, even though the authors of that study describe “Enhanced competence”, they do not describe aspects of “Professional comfort when dealing with moral challenges” as presented in this study. Finally, they describe “Improving quality of care” in a similar way to the “*Improved quality of care for the child and the family*” theme in this study.

In previous research on CESS outcomes, HCPs acknowledge that dealing with interprofessional uncertainty and different viewpoints within MCDs can promote interprofessional understanding, leading to reduction of conflicts in care initiatives [16, 26]. In turn, reduction of conflicts may help to enhance the sense of security in the interprofessional team. It is important to consider that participants’ desire for security could be related to cultural reasons and may differ between contexts and countries.

The results of this study show a perceived need for improved teamwork. The importance of interprofessional teamwork has also been highlighted in previous CESS research [26, 34–36]. This result further underscores the importance of teamwork in all parts of healthcare where the collaborative contribution of several professionals is important in order to provide high-quality care. MCDs for interprofessional team reflection will be implemented after this study, and it will be interesting to see whether there is any change in the experience of the quality of interprofessional teamwork as a result.

In this study, *being confirmed* was perceived as an important outcome of MCDs. *Being confirmed* meant that participants’ thoughts and opinions were acknowledged by the interprofessional team. The results of a study on reflective teams and supervision in healthcare indicate that if the team maintains an open atmosphere, moral judgements can be avoided, and internal demands can be replaced by confirmation [37]. Furthermore, Friberg et al. (2016) suggest that when HCPs do not acknowledge each other’s views and feelings, there is a risk of feeling uncertain, experiencing a lack of confidence and increased feelings of doubt in their own profession [38]. The results of our study, as well as those of another study on evaluation of CESS [34], show that HCPs feel it is important to strengthen their self-confidence when managing morally challenging situations. In our study it was found that one important aspect of strengthening self-confidence is to be confirmed. By this, we suggest that confirmation is needed in order to handle moral uncertainty and doubt. However, research relating to moral challenges in healthcare shows that feelings of uncertainty are all part of daily life [39]; or, more precisely, that moral uncertainty is an inherent part of working in paediatrics. Learning to handle moral uncertainty is therefore an important part of developing professionalism and dealing with moral challenges. This raises the question of the extent to which MCDs could contribute to confirming HCPs in order to reduce their feelings of moral uncertainty and insecurity in childhood cancer care.

Developing moral courage was perceived as an important MCD-related outcome and is thematised in the analyses as part of “Professional comfort when dealing with moral challenges”. Feelings of professional uncertainty may result in a sense of vulnerability when providing care and within the interprofessional team, in the sense that HCPs underestimate themselves and feel unprofessional. Vulnerability is related to courage: feeling uncertain and asking for help is an example of a situation in which we feel vulnerable and require courage [40]. Furthermore, vulnerability and courage may affect any relationship, particularly in care and interprofessional relationships in childhood cancer care, where HCPs are exposed to emotionally difficult situations on a daily basis. It may be claimed that if HCPs have the ability to acknowledge vulnerability and feel courageous, they will be more able to cope with the emotionally difficult situations arising from relationships with the recipients of care. Moral courage also appears to create both energy and strength that improves HCPs’ sense of hope and responsibility in their work [41]. One challenging question relates to how HCPs can express their vulnerability and share their experience with their interprofessional team during MCDs, particularly when those MCDs take place in what are sometimes quite hierarchical healthcare teams [27, 42]. A more methodological question is what HCPs really meant when they stated that they found it important to develop courage; could they have meant developing confidence? In the literature, being courageous is described as a virtue and a characteristic that is associated with a moral approach that preserves the patient’s dignity by considering ethical values ​​such as respect and responsibility [43, 44]. In addition, moral courage in nursing is also strongly associated with the professional role such as personal risks, commitment and true presence [45]. It would be interesting to explore more deeply the meaning of moral courage in MCDs, and to study whether moral courage can be further developed through participation in MCDs.

Improved quality of care for the child and the family includes understanding the child’s specific situation and needs. Previous research into barriers to clarifying perspectives in MCDs highlights that unsuccessfully understanding the child’s current situation might risk failing to consider the viewpoints of the patient and their family [27]. The fact that participants in this study found it important for MCDs to lead to improvements in the quality of childhood cancer care, including patient safety, needs further exploration. For example, if MCDs help to enhance teamwork, this might reduce the risk of medical errors being made [46]. Other evaluation studies do indeed seem to suggest that CESS improves the quality of care [47, 48]; yet few studies show whether this is actually the case and what specific CESS outcomes actually contribute to quality of care, and in what way [49, 50]. It would be very interesting to explore this further by conducting in-depth interviews with HCPs who have participated in MCDs; for example, asking them about their perceptions of the impact of MCDs on quality of childhood cancer care.

As mentioned in the introduction, the aim of this study was to search for context-specific results. In this study, we were able to gather information from HCPs in all six Swedish childhood cancer care centres about the importance of MCD-related outcomes. As a result, the subsequent implementation of MCDs in each of these centres can be tailored to HCPs’ needs. The study results can also be used for the training of MCD facilitators in all Swedish childhood cancer care centres, as MCD facilitators will know which specific MCD-related outcomes are most appreciated. This will enable MCD facilitators to structure and guide the sessions in a way that promotes the realisation of the desired outcomes. Furthermore, managers from the centres and the HCP teams may make use of the information about which outcomes are deemed important according to their interprofessional teams. This might help them with implementing MCDs and monitoring specific outcomes. The results of this study may also be useful for managers in that the study underscores the importance of several aspects of teamwork for good clinical care, which could be enhanced in other ways than through MCDs, such as general work on attitudes in the clinic, meeting forums and education.

### Limitations and strengths of this study

One possible limitation of this study is that the open-question design resulted in some short answers, with no possibility for subsequent clarification. Some of these short answers were difficult to analyse and interpret; for example, what was meant by the word ‘professionalism’. Even if the data consists of some short sentences, we would argue that the data is still relevant, in particular in a CESS outcomes study since there is little research about what MCD-outcomes HCPs themselves envision when MCDs are implemented. Instead of investigating outcomes based on responses to predefined lists by ethicists/researchers, the present study explores inductive perceptions of HCPs, giving HCPs a voice and ownership of CESS.

One interesting aspect of the study is how participants interpreted the open-ended question. Some participants answered in relation to MCD outcomes achieved during the process, while others thought in terms of outcomes achieved after the MCD. Different interpretations of the open-ended question were also found in the Dutch study [34]. However, we would argue that regardless of what participants were aiming for (during or after), the described outcomes are valuable to support HCPs in handling moral challenges; and more specifically, to offer HCPs relevant and empirically requested CESS in the provision of childhood cancer care.

A central strength of the present study is that it is a national study involving all childhood cancer care centres in Sweden, and various professional groups, with a satisfactory response rate of almost 60%. In this way, the study not only provides specific insights for the domain of childhood cancer care, but also generates insights at a national level. Furthermore, the relevance of this data is high, given the fact that follow-up evaluation research will be carried out at each centre, using the Euro-MCD instrument (part II). The follow-up evaluation research will make it possible to compare the results of this study with future evaluation studies, and to determine whether the MCD outcomes that HCPs found most important have indeed been realised and effectuated. Finally, another strength of this study is investigator triangulation, whereby researchers from different countries and childhood cancer centres [31] worked together to create a broad analytical space and contributed to bring about enhanced insights [31]. This triangulation is also an appropriate method for validating the research process, and it minimises the risk of misinterpretation and possible bias [51].

## Conclusion

Before implementing MCDs, it is important to know what outcomes are considered important by future participants. HCPs working in childhood cancer care in Sweden answered questions about what MCD outcomes they found important, with no explicitly declared internal hierarchy, prior to their participation in MCDs. Themes of important outcomes were: *Interprofessional well-being in team interactions; Professional comfort when dealing with moral challenges;* and *Improved quality of care for the child and the family,* indicating that HCPs have high expectations of and strong belief in MCDs. One particularly interesting finding was that HCPs stressed the importance of outcomes related to feeling secure, brave and confirmed. The results of this study can be used in order to monitor and foster MCDs as well as during the implementation of MCDs in Swedish childhood cancer care centres. Furthermore, this context-specific research may also constitute a foundation for the design of future outcomes studies in paediatric CESS. In addition, the findings of this study will be helpful in current and future training of MCD facilitators in childhood cancer care. Facilitator training could be adapted based on the knowledge of context specific important MCD outcomes. Managers making decisions about the implementation of CESS would also benefit from knowing about the specific important outcomes for their target group.

## Data Availability

The datasets used and/or analysed during the current study are available from the corresponding author on reasonable request.

## Abbreviations

CESS: Clinical ethics support services
HCP: Healthcare professional
MCD: Moral case deliberation
